# Antioxidant Protection from UV- and Light-Stress Related to Carotenoid Structures

**DOI:** 10.3390/antiox8070219

**Published:** 2019-07-11

**Authors:** Gerhard Sandmann

**Affiliations:** Institute of Molecular Biosciences, Goethe-University Frankfurt/M, Max von Laue Str. 9, D-60438 Frankfurt, Germany; sandmann@bio.uni-frankfurt.de

**Keywords:** anti-oxidants, carotenoids, lipid peroxidation, light-stress, radicals, singlet oxygen, structure activity relationship, UV-stress

## Abstract

This review summarizes studies of protection against singlet oxygen and radical damage by carotenoids. The main focus is on how substitutions of the carotenoid molecules determine high antioxidant activities such as singlet oxygen quenching and radical scavenging. Applied assays were carried out either in vitro in solvents or with liposomes, and in a few cases with living organisms. In the latter, protection by carotenoids especially of photosynthesis against light- and UV-stress is of major importance, but also heterotrophic organisms suffer from high light and UV exposure which can be alleviated by carotenoids. Carotenoids to be compared include C_30_, C_40_ and C_50_ molecules either acyclic, monocyclic or bicyclic with different substitutions including sugar and fatty acid moieties. Although some studies are difficult to compare, there is a tendency towards mono and bicyclic carotenoids with keto groups at C-4/C-4’ and the longest possible polyene structure functions to act best in singlet oxygen quenching and radical scavenging. Size of the carotenoid and lipophilic substituents such as fatty acids seem to be of minor importance for their activity but hydroxyl groups at an acyclic end and especially glycosylation of these hydroxyl groups enhance carotenoid activity.

## 1. Oxidants and Antioxidants

Since the accumulation of oxygen in the atmosphere around 2 billion years ago caused by oxygenic photosynthesis, all living organisms have to cope with oxidative stress. Especially the reactive oxygen species (ROS), such as singlet oxygen ^1^O_2_, hydroxyl radicals HO•, superoxide anion radicals O_2_**^•^**^−^ and hydrogen peroxide H_2_O_2_, generated by photosensitization or in cellular reactions exert their destructive power on the metabolism. Targeted metabolites are amino acids in enzymes, nucleic acids and fatty acids in lipid membranes [1]. ^1^O_2_ is a very strong oxidant. It represents the electronically excited state of molecular oxygen with no unpaired electrons and is formed by energy transfer to the ground state from an excited photosensitizer. Photosensitizers are cell specific compounds such as porphyrins, riboflavin, chlorophylls or UV absorbing molecules [2]. The most efficient part of the solar spectrum is blue light and UV-B and UV-C radiation. 

**Figure 1:**
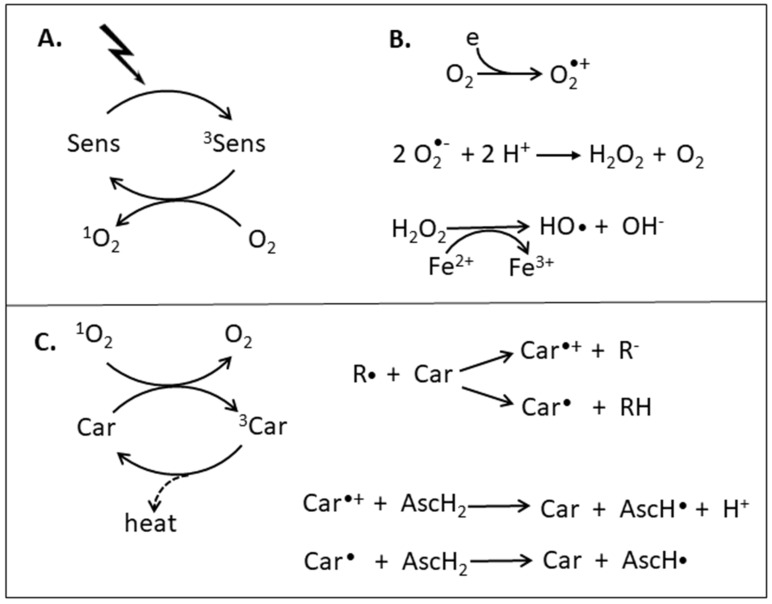
Formation of reactive oxygen species and their reaction with carotenoids. (**A)** Photosensitized formation of singlet oxygen, (**B**) radical formation, (**C**) reactions of carotenoids with singlet oxygen or radicals and regeneration by ascorbate.

In the course of evolution, organisms have developed several strategies to protect from ROS [3]. They include enzymatic reactions to split H_2_O_2_ and antioxidants for hydrophilic (e.g., ascorbic acid) or lipophilic regions within the cell to inactivate HO• or quench ^1^O_2_. The most prominent lipophilic antioxidants are tocopherols (vitamin E) and carotenoids. The latter pigments are also able to absorb the radiation energy from the photosensitizer preventing the transfer of excitation energy to ground state oxygen (Figure 1A). This is one way to prevent accumulation of ^1^O_2_. Since the triplet energy level of carotenoids is close or below that of ^1^O_2_, they can also efficiently drain the excitation energy from ^1^O_2_. This absorption of excitation energy and its dissipation as heat is the principal mechanism of carotenoids to protect organisms from photosensitized formation and accumulation of ^1^O_2_ [2,4].

A prominent function of carotenoids is the protection of the photosynthesis apparatus from damage under high light conditions [5]. This includes quenching of photosensitized triplet chlorophyll as first line of defense and of single oxygen once formed by reaction with triplet chlorophyll (Figure 1B). Another consequence of excess light is the formation of superoxide anion radicals O_2_•^−^ by reduction of O_2_ in the photosynthetic electron transport chain. Disproportionation of O_2_•^−^ is a source of H_2_O_2_ (Figure 1B) [6]. Radicals such as O_2_•^−^ and others including hydroxyl radical HO• which are formed from H_2_O_2_ by a Fenton-type reaction with Fe^2+^ or Cu^+^ [7] can be inactivated by carotenoids. Several mechanisms are discussed by which carotenoids interact with radicals (Figure 1C). One type of reaction is adduct formation R-Car• by radical addition to the carotenoid polyene chain [8], another involves removal of an electron from the conjugated system of the carotenoid by the radical leading to the formation of a carotenoids radical cation Car•^+^ with a delocalized unpaired electron [2,9]. A third possibility is hydrogen abstraction from especially from allylic positions of the carotenoid yielding the radical Car• [10]. Finally, the carotenoid can be regenerated from a resonance-stabilized carotenoid radical by reaction with different reductants which is best studied for ascorbate [11,12]. In addition to radical scavenging by carotenoids as protective reaction, radical-carotenoid adducts are able to form peroxyl radicals under high oxygen pressure. Subsequently, the peroxyl radical acts as a generator of radicals in radical chain reactions involving for example oxidation of fatty acids in lipids [13,14]. However, this pro-oxidant reaction of carotenoids is minor under physiological oxygen concentrations. Not only in photoautotrophic but also in heterotrophic organisms, carotenoids are synthesized as antioxidants to protect especially against UV-generated ROS.

## 2. In Vitro Antioxidant Assays for Different Carotenoids

Several hundreds of carotenoid structures are known; they differ in their carbon chain lengths, the conjugated double bond system and the presence or absence of ionone rings with different substitutions [15]. Over the decades, their properties as radical scavengers and ^1^O_2_ quenchers in relation to their structures were investigated. Experiments to determine their function in artificial systems were facilitated by the availability of novel carotenoids isolated from microorganisms or by their combinatorial synthesis through genetic engineering [16]. Furthermore, genetic modification of the carotenoid composition within organisms allowed the study of their functionality directly in living cells. 

The applied assay systems for ^1^O_2_ quenching or radical scavenging use different photosensitizers or radical generators, respectively, whereas ^1^O_2_ or radical concentrations were measured directly or by oxidation of different substrates. This makes it difficult to compare the results and to rank the tested carotenoids [2]. Application of the carotenoids in the assays may be in fixed concentrations resulting in relative protection or by determination of the concentration values responsible for 50% quenching compared to a control without added carotenoid (IC_50_). The assays can also be focused on ^1^O_2_ formation versus ^1^O_2_ peroxidation or radical formation versus peroxidation by radicals [17].

### 2.1. O_2_ Quenching by Carotenoids

**Figure 2:**
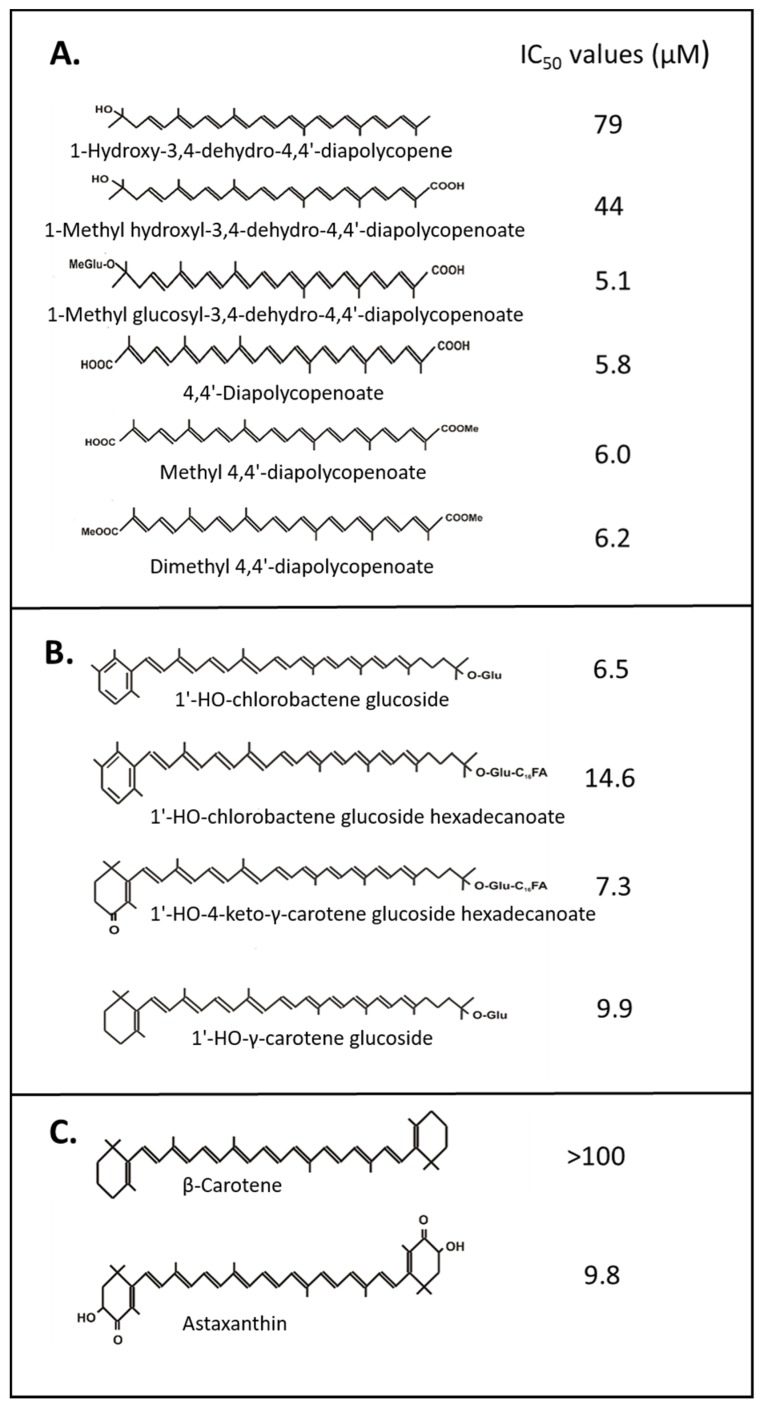
IC_50_ values of carotenoids as ^1^O_2_ quenchers assayed according to [20]. (**A**) C_30_ 4,4’-diapolycopene derivatives [20,21,22,23], (**B**) monocyclic carotenoids [24], (**C**) astaxanthin and β-carotene for comparison [23].

Most data on ^1^O_2_ quenching were obtained with toluidine blue or methylene blue with similar chromophores as photosensitizers, and linoleic acid as the substrate to monitor ^1^O_2_ formation. In this type of system, 18 cyclic and acyclic C_40_-carotenoid structures differing in the number of conjugated double bonds and oxy group substitutions were investigated [18]. The authors show that the strongest protection effect is exerted with increasing numbers of conjugated double bonds. A smaller effect could be attributed to HO-groups and a major one to keto groups which extend the conjugated system as in astaxanthin. In a similar assay, several authors determined IC_50_ values for carotenoids to suppress ^1^O_2_ accumulation. A comparison of the values for astaxanthin and its fatty acid esters gave no conclusive results. Either differences were very small [16] or the results varied with the use of the solvents [19]. In contrast to 100% ethanol, the IC_50_ was much higher in 50% hexane than in ethanol. The reason for the lower effectiveness of astaxanthin may be its poor solubility in the latter solvent mixture. Comparable IC_50_ values were also determined for a set of C_30_ 4,4′-diapocarotenoids [20,21,22,23] in comparison with the poorly protective C_40_ β-carotene and highly protective astaxanthin (Figure 2). The data show that C_30_ carotenoids although shorter are also efficient ^1^O_2_ quenchers. Their antioxidant properties also gradually increase with the extension of the polyene chain from 10 via 11 to 13 conjugated double bonds including both keto groups in 4,4’-diapolycopendioc acid derivatives. Formation of its monoester decreased the quenching activity which was lowest with the diester. When comparing monocyclic carotenoids either with a β-ionone or a φ-ionone (= aromatic) ring, the aromatic end group enhanced ^1^O_2_ quenching, whereas a C_16_ fatty acid bound to the sugar decreased this activity (Figure 2B) [24].

**Figure 3:**
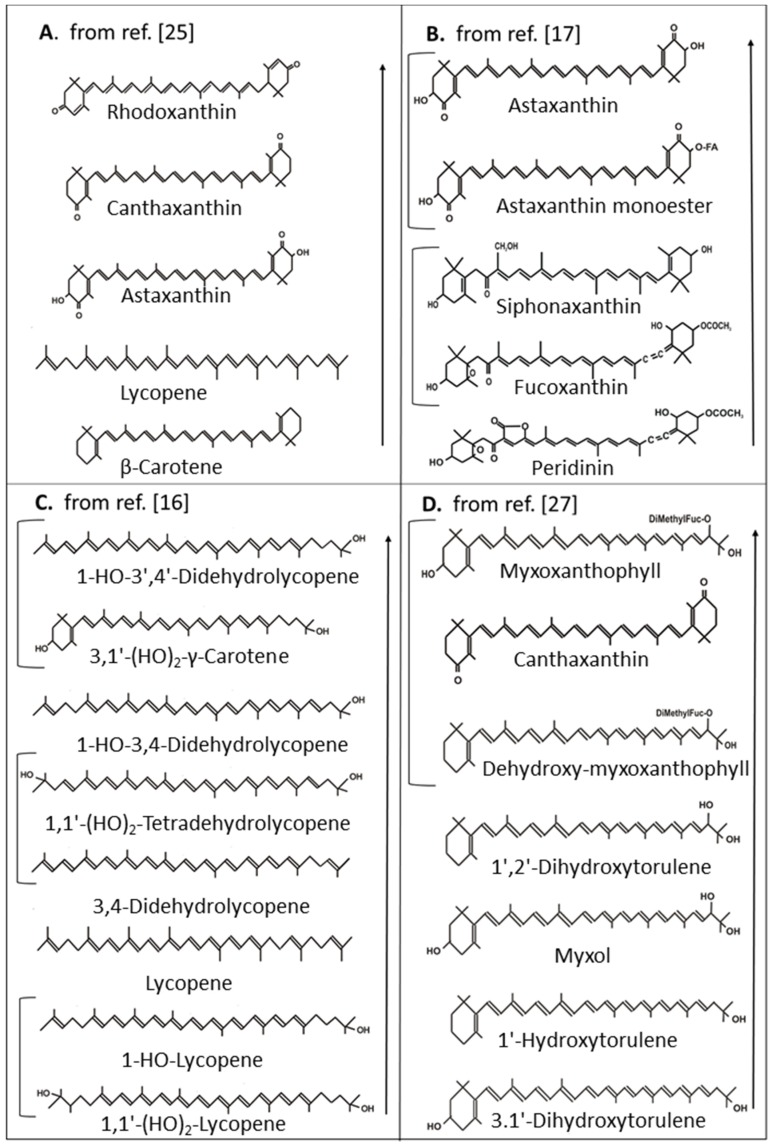
Comparison of ^1^O_2_ quenching activities of different carotenoids. Arrows indicate increasing activity, brackets indicate similar activity.

In an extensive study with chemically synthesized carotenoid analogues, their protective function on accumulation of chemically generated ^1^O_2_ was investigated [25]. Among the natural carotenoids, rhodoxanthin with the same C_40_ carbon backbone and similar substitutions as canthaxanthin, but with 3,3’-diketo groups and 14 instead of 13 conjugated double bonds in retro configuration, exhibited the highest protection, followed by canthaxanthin, astaxanthin, lycopene and β-carotene (Figure 3A). It is notable that although naturally existing astaxanthin is a much better ^1^O_2_ quencher than β-carotene, the synthetic C_50_ hydrocarbon decapreno β-carotene is more protective than the C_50_ astaxanthin analog. Results completely different to the studies mentioned above were obtained by Di Mascio et al. [26] with chemically generated ^1^O_2_ and its quenching by carotenoids measured directly by infrared emission of ^1^O_2_. Under these test conditions, lycopene, although with a shorter polyene system, was superior to astaxanthin and also γ-carotene was as effective as astaxanthin. Also, lutein, an α-carotene derivative with two hydroxyl groups, exhibited lower quenching than the unsubstituted hydrocarbon. 

Apart from assays determining protection by carotenoids on initial or early events of photosensitized peroxidation, other approaches focused on photosensitized reactions with artificial membranes. For a series of keto carotenoids, a comparison of both methods was made [17] showing that both provide equivalent results. Compared to astaxanthin with 13 conjugated double bonds with best protection, siphonaxanthin with 9 conjugated double bonds was less protective and fucoxanthin and peridinin with 8 plus one additional allenic double bond quenched even less (Figure 3B). Differences in their activity must be due to the different modification of the central carbon chain. Another study with the artificial membrane system compared lycopene with its hydroxyl derivatives and further desaturated polyene chains [16]. Among the carotenoids with the best protection against ^1^O_2_ was 1-HO-3’,4’-didehydrolycopene with 13 conjugated double bonds and one hydroxyl group at C-1 followed by 1-HO-3,4-didehydrolycopene with 12 conjugated double bonds and the same hydroxyl group (Figure 3C). 3,4-Didehydrolycopene and 1,1’-(HO)_2_-tetradehydrolycopene with 13 conjugated double bonds but differing from 1-HO-3’,4’-didehydrolycopene by the lack of the hydroxyl group or the presence of an additional one at C-1′ exhibited an even lower activity. Low activity in protection against ^1^O_2_ was observed with unsubstituted lycopene which even gradually decreased with the presence of one or two hydroxyl groups at C-1 and C-1’. The only monocyclic carotenoid in this series was 3,1’-(HO)_2_-γ-carotene with 11 conjugated double bonds and hydroxyl groups at C-3 of the β-ionone ring and at C-1 which had the same ^1^O_2_ quenching potential as 1-HO-3’,4’-didehydrolycopene with 13 conjugated double bonds.

In photosynthetic organisms, chlorophyll is the dominant photosensitizer. Therefore, protection against chlorophyll-sensitized membrane oxidation was investigated with a set of monocyclic carotenoids together with canthaxanthin and lycopene for comparison [27]. Best protection was by canthaxanthin and both glycosides myxoxanthophyll and dehydroxy-myxoxanthophyll differing only by a 3-hydroxyl group (Figure 3D). Next in line with lower activity was 1’,2’-dihydroxytorulene the aglycone of dehydroxy-myxoxanthophyll followed by myxol the aglycone of myxoxanthophyll together with 1’-hydroxytorulene resembling 1’,2’-dihydroxytorulene without the 2’-hydroxyl group and 3,1’-dihydroxytorulene resembling the structure of 1’-hydroxytorulene without the 3-hydroxyl group. Lycopene was not protective in this series.

### 2.2. Radical Scavenging by Carotenoids

Radical scavenging by carotenoids was assayed with a radical generator either directly without a substrate (radical formation assay) or with the addition of phosphatidylcholine (PC) liposomes for lipid peroxidation (radical peroxidation assay). Radical generators used were either water soluble compounds such as 2,2’-azo-bis(2-amidinopropane) hydrochloride (AAPH) and 2,2’-azino-bis(3-ethylbenzothiazoline-6-sulphonic acid (ABTS) or lipid-soluble ones such as 2,2’-azo*bis*(2,4’-dimethylvaleronitrile (AMVN), 2,2-azo-*bis*-isobutyronitrile (AIBN) and di(phenyl)-(2,4,6-trinitrophenyl) imino azanium (DPPH). 

One investigation compares the scavenging activities in the radical formation assay versus the radical peroxidation assay with a set of algal keto carotenoids [17]. Especially fucoxanthin and peridinin both with an allenic double bond were much more efficient in protecting from peroxidation with ABTS cation radical than preventing lipid peroxidation initiated by DPPH, a stable free radical, whereas astaxanthin, its fatty acid monoester and siphonaxanthin inhibited well in both assays. 

**Figure 4:**
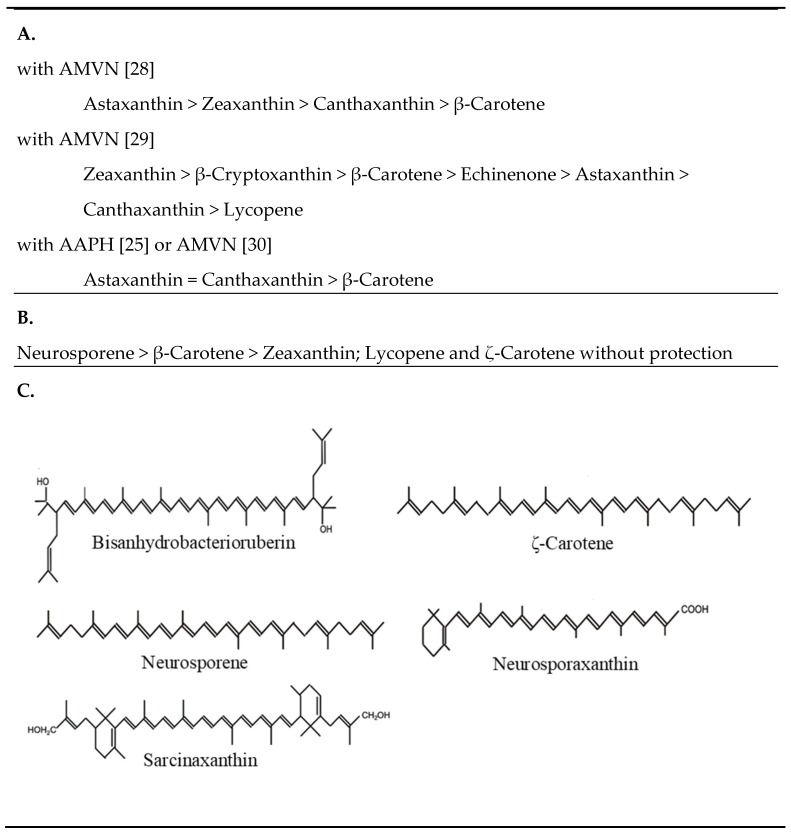
Protection by carotenoids against peroxyl radicals (**A**), against UV with α-terthienyl as the sensitizer (**B**), carotenoid structures mentioned in Section 3.1 and not shown in other figures (**C**). AMVN: 2,2′-azo*bis*(2,4’-dimethylvaleronitrile; AAHP: 2,2’-azo-bis(2-amidinopropane) hydrochloride.

Radical peroxidation assays in the presence of phosphatidyl choline liposomes were carried out by several authors with different carotenes and hydroxylated and ketolated β-carotene derivatives. This included protection against radical initiation with AAPH or AMVN. Lipid-soluble AMVN generates peroxyl radicals by thermolysis which resembles the biological situation best. A study on scavenging with AMVN by carotenoids [28] showed that astaxanthin was superior to all other carotenoids tested followed by zeaxanthin, then canthaxanthin and finally β-carotene (Figure 4A). Under similar conditions but at a higher rate of peroxyl radical formation caused by a higher temperature, the order of carotenoids was not consistent [29] since here zeaxanthin and then β-cryptoxanthin were most efficient followed by β-carotene whereas the keto derivatives, echinenone, astaxanthin and canthaxanthin protected less (Figure 4A). However, a high radical trapping by zeaxanthin and canthaxanthin and poor activity of β-carotene corresponds well to carotenoid oxidation with AAPH [30] or with HO• generation in a Fenton reaction by H_2_O_2_ and reduced iron [10]. Independent of the type of radical generation, carotenoid radical scavenging in lipid peroxidation is synergistic with other antioxidants such as α-tocopherol [29].

The same lycopene derivatives tested for ^1^O_2_ quenching were also assayed for protection of radical lipid peroxidation started by AIBN [16]. The order of radical scavenging was the same as for the inactivation of ^1^O_2_ with the exception of 1,1’-(HO)_2_-tetradehydrolycopene which showed the lowest scavenging activity apart from 1,1’-(HO)_2_-lycopene.

For the C_50_ bacterioruberin IC_50_ values for quenching of DPPH radical signal demonstrated that this carotenoid is 3-fold better in radical inactivation than β-carotene [31].

## 3. Protection in Living Organisms by Carotenoids against High Light and UV Radiation

Protection especially of the photosynthetic apparatus against photo oxidation and the formation of ROS is essential for autotrophic organisms. Nevertheless, also non-photosynthetic organisms are susceptible to high light and UV radiation and use carotenoids for protection [32]. Antioxidant activities of carotenoids can be tested in living organisms by quantitative or qualitative variation of their carotenoid composition. In animal studies, carotenoids such as β-carotene, canthaxanthin or astaxanthin injected or fed with the diet exerted antioxidant activity (see [33] for an overview). In microorganisms, testing of carotenoids can be better achieved by use of pigment mutants, inhibitors of carotenoid biosynthesis and genetic pathway modification followed by enhancement of oxidative stress either by application of a photosensitizer or by an oxidant.

### 3.1. Heterotrophic Organisms

One of the first studies to demonstrate carotenoid protection against photodynamic damage was carried out with *Micrococcus luteus* (formerly *Sarcina lutea*), a bacterium containing the C_50_ carotenoid sarcinaxanthin [34] as well as with mutants with lower or zero carotenoid content, by direct sunlight exposure [35] or after adding a photosensitizer [36]. In each case, the number of survival cells of the non-pigmented mutant was orders of magnitude lower than the pigmented wild type. Similar results were obtained with *Curtobacterium flaccumfaciens* (formerly *Corynebacterium poinsettiae*) containing C_50_ bisanhydrobacterioruberin [37] after inhibition of carotenogenesis and with its non-pigmented mutant. This approach also demonstrated that the action of the photosensitizer was oxygen dependent [38]. C_30_ 4,4’-diapolycopene-dioic acid ester also protects growth of a *Bacillus* species against peroxide-induced peroxidation [39].

Carotenoids are known to protect against UV-B radiation in fungi, but they can also play a role as substrates for formation of cleavage products with functions in fungal metabolism. Treatment of strains of *Ustilago violacea* with visible and UV radiation showed that carotenogenic strains containing neurosporene and lycopene [40] were more resistant to cell death caused by light, demonstrating the effectiveness of carotenoids as protectants [41]. Similar studies carried out with the neurosporaxanthin-synthesizing fungus *Neurospora crassa* gave comparable results [42]. The importance of carotenoids in photoprotection is supported by the light-dependent up-regulation of the synthesis of these pigment in many fungal species [43].

Non-carotenogenic *Escherichia coli* is susceptible to near UV radiation [44]. Transformants carrying a plasmid for the synthesis of zeaxanthin diglycoside had a much higher survival rate. The protection by this carotenoid was much stronger when UV treatment was carried out in the presence of the UV-sensitizer α-terthienyl [45]. When *E. coli* was engineered to synthesize different carotenoids in a similar concentration range, the carotenes neurosporene followed by β-carotene were the most protective against UV treatment but also zeaxanthin showed some degree of protection unlike lycopene and ζ-carotene, especially when oxidative pressure was increased with α-terthienyl (Figure 4B) [46]. Zeaxanthin acted concentration dependent and zeaxanthin diglycoside was less effective even in higher concentrations.

Oxidative stress in humans and health benefits of carotenoids were reviewed recently [47]. Extended exposure of light causes skin damage in animals. Photosensitivity of the skin is strongly pronounced in light-sensitive porphyria in which porphyrin is accumulated as an endogenous photosensitizer triggering the generation of ^1^O_2_. In an experiment with albino mice, the application of hematoporphyrin and exposure to black light with short UV radiation phases was lethal to most individuals [48]. Injection of β-carotene prior to treatment resulted in a significantly higher number of surviving animals. Patients with erythropoietic protoporphyria suffer from a disturbed porphyrin metabolism causing an accumulation of protoporphyrin in the body. This makes the skin extra susceptible to photosensitation. Indigestion of a α- and β-carotene diet over several weeks resulted in an improved tolerance for light with a suppression of the burning symptoms [49]. These results demonstrate that in vivo carotenoids can alleviate skin damage caused by porphyrin photosensitation by quenching either its exited state or the resulting ^1^O_2_. However, a high dose of β-carotene tested on isolated keratinocytes from the skin led to pro-oxidant action [50]. This pro-oxidant effect demonstrated in an animal model with high β-carotene application in combination with smoke exposure may also be responsible for increased risk for smokers of lung cancer when treated with high carotenoid doses [51].

### 3.2. Photosynthetic Organisms

In diatoms, fucoxanthin is the major carotenoid of the chloroplast. By inhibition of its synthesis to about half the concentration of the control, the alga became highly susceptible to high light and peroxide treatment [17]. In photosynthetic algae and plants, carotenoids in the chloroplast not only function as antioxidants but are also constituents of pigment protein complexes functioning in photosynthetic electron transport and especially in light-harvesting [52]. In plant photosynthesis, carotenoids are not only protective but also act as accessory pigments for light-harvesting. This light-harvesting function is replaced in cyanobacteria by phycocyanin [53]. Therefore, for investigations focusing on carotenoid antioxidant action in oxygenic photosynthesis, cyanobacteria are the most useful organisms since modification of carotenoid composition avoids disturbance of light-harvesting protein complexes and their function. Consequently, carotenoid action can be mostly attributed to protection of photosynthesis or chlorophyll levels during the influence of light or UV radiation. 

The unicellular canobacterium *Synechocystis* synthesizes a combination of myxoxanthophyll, β-carotene, echinenone and zeaxanthin. By inactivation of two genes of the carotenoid pathway, deletion mutants have been generated which lack either echinenone formation, zeaxanthin formation or the synthesis of both carotenoids, simultaneously. With these mutants, the effects of high light inhibition of photosynthetic activity and methylene blue sensitized chlorophyll oxidation were determined [54]. Lack of echinenone had only a moderate effect on both parameters, but lack of zeaxanthin strongly decreased photosynthesis and chlorophyll content. When both carotenoids were missing, *Synechocystis* suffered considerably more. *Synechococcus* is another unicellular cyanobacterium which only accumulates β-carotene and zeaxanthin. By transformation, its zeaxanthin content was either increased or converted to canthaxanthin [55,56]. The influence of the modified carotenoid composition on photosynthesis under high light or treatment with near UV is shown in Table 1. High zeaxanthin in the cells prevented a decrease of photosynthesis in high light as well as under UV. Prevention of UV damage was directly correlated to the zeaxanthin content of the transformants. A replacement of zeaxanthin by canthaxanthin almost completely protected photosynthesis in high light. From the investigations with these cyanobacteria, it can be concluded that of the carotenoids present in cyanobacteria, canthaxanthin is the best protectant of photosynthesis against high light and UV radiation followed by zeaxanthin and then by echinenone. The significance of these carotenoids in the protection of photosynthesis in cyanobacteria is supported by the light-dependent up-regulation of canthaxanthin synthesis [57] or zeaxanthin synthesis [58] in different species. In plant photosynthesis, the major protecting carotenoid is zeaxanthin which is generated by de-epoxidation of violaxanthin in the xanthophyll cycle under high light conditions. Due to the UV defense attributed to zeaxanthin, tobacco plants were genetically engineered to synthesize zeaxanthin in larger quantities [59]. Upon treatment with UV or ^1^O_2_ generated by the dye rose bengal as a photosensitizer, a decrease in photosynthetic oxygen evolution was lower and lipid peroxidation less pronounced than in the wild-type with lower zeaxanthin content. With tobacco as a higher plant model, it could be demonstrated that enhanced zeaxanthin protects photosynthesis under radiation stress leading to a higher biomass.

## 4. Conclusion on Carotenoids as Antioxidants

The antioxidant function of carotenoids as quenchers of excited photosensitizers and ^1^O_2_, as well as their radical scavenging potential, is well-documented. The degree of these activities depends on their chemical structures. The in vitro assays to assess their antioxidant potential can be divided into those targeting ^1^O_2_ formation and peroxidation by ^1^O_2_ or radical formation and peroxidation by radicals. Among the carotenoids for which IC_50_ values for ^1^O_2_ inactivation are available, substituted acyclic C_30_ carotenoid diacids showed the highest activities (Figure 2A). In general, superior carotenoids for ^1^O_2_ quenching are those with the longest polyene system corresponding to the lowest triplet energy levels which is especially the case for rhodoxanthin (Figure 3A) and for HO-chlorobactene glucoside with an aromatic end group (Figure 2B). Substituents affect the electronic density in the polyene chain. There is no clear tendency of electron withdrawing hydroxyl groups at acyclic carotenoids (Figure 3C). They negatively affect ^1^O_2_ quenching activity of acyclic carotenoids (Figure 3C) but seem to be favorable when located at α-position to the polyene chain of monocyclic carotenoids (Figure 3D). Hydroxyl groups at C-3 of the β-ionone ring somehow decrease quenching activity. However, glycosylation of a hydroxyl group strongly enhanced ^1^O_2_ quenching (Figure 3D). When the sugar moiety is esterified with a fatty acid, activity decreased again (Figure 2A,B). In a similar way, esterification of the carboxylic groups of the C_30_ carotenoids lowers the quenching activity (Figure 2A). The comparison of dihydroxy-γ-carotene to dihydroxylycopene both with 13 conjugated double bonds indicated that a monocyclic structure is more favorable for ^1^O_2_ quenching than an acyclic structure (Figure 3C). An important substituent at the β-ionone rings for high protection against ^1^O_2_ are keto groups at positions C-4 and C-4’ which extend the conjugated double bond system (Figure 3A,B). Only a very few studies demonstrating ^1^O_2_ quenching by carotenoids in non-photosynthetic organisms are available. Transgenic *E. coli* synthesized varying carotenoids. The ^1^O_2_ quenching activity of these transformants differed fundamentally from the in vitro results. In this bacterium, neurosporene with only 9 double bonds protected best and better than lycopene, and even zeaxanthin, against photosensitized UV treatment (Figure 4B). However, in cyanobacteria with oxygenic photosynthesis, protection of different carotenoids against light and UV damage in general resembled the carotenoid activities of the in vitro results (Table 1). 

Substantial radical scavenging in vitro was obtained with most of the carotenoids which also protect against ^1^O_2_. One way for natural carotenoids to react with radicals is by hydrogen abstraction from the allylic carbon such as C-3 in canthaxanthin and C-2 in rhodoxantin or from the 3-hydroxyl group of astaxanthin. This ensures the best resonance stabilization of the resulting carotenoid radical due to the extended polyene system allowing for delocalization over most of the carotenoid molecule. For zeaxanthin with the allylic carbon at C-4, the polyene system is shorter and radical delocalization less pronounced corresponding to a somehow lower radical inactivation activity. In the case of electron capture by a radical from the carotenoid (Figure 1C), the polyene chain is as important for the resonance stabilization of the resulting carotenoid radical cation as for the carotenoid radical generated by H• abstraction.

Either for ^1^O_2_ quenching or radical inactivation, the length of the carotenoid seems of minor importance since among C_30_, C_40_ and C_50_ derivatives highly potent species can be found. Polar sugar moieties or carboxylic groups at the end of the carotenoid molecule enhance quenching activity (Figure 3B) whereas fatty acid substituents at the sugar reverse this effect (Figure 2A,B). This points at an advantageous anchoring with these polar groups to the hydrophilic outside of the membrane [60] but with a free floating tail in the hydrophobic core of the membrane. However, the best tested ^1^O_2_ quenching carotenoid is the synthetic C_50_ hydrocarbon decapreno β-carotene without any polar groups [25] which should be completely mobile in the membrane. Therefore, positioning and orientation of carotenoids in the lipid membrane seems to be less crucial for their antioxidant function than generally discussed [29]. 

The in vitro studies provide detailed information on the structural properties of carotenoids which are responsible for maximum protection against ^1^O_2_ formation and inactivation and for making them good radical quenchers. Only a few investigations on antioxidant action of carotenoids in bacteria and fungi are available. They demonstrate that carotenoids protect against radiation and oxidative stress but are not sufficient to recognize optimized structures. In photo autotrophic organisms, damage on photosynthesis is mainly caused by excited chlorophyll leading to formation of ^1^O_2_. By genetic modification of the carotenoid composition it was evident that diketo β-carotene (canthaxanthin) is superior to dihydroxy β-carotene (zeaxanthin) and that β-carotene is of minor importance (Table 1). These results correspond well to those obtained in vitro with chlorophyll as a photosensitizer (Figure 3D).

## Figures and Tables

**Table 1:** Protection of photosynthetic oxygen evolution in *Synechoccocus* transformants with increased zeaxanthin or canthaxanthin content after exposure to high light or UV-B [55,56].

Transformants	Carotenoid (mg/g dw)	Photosynthesis (% of Untreated Control)	
		High Light *	UV Treatment **
non-transgenic	Zeax 1.0	66	32
CrtB	Zeax 1.1	--	37
Psy	Zeax 1.7	--	58
CrtZ	Zeax 1.9	82	60
Bkt ***	Canth 1.6+ Zeax 0.7	95	--

* 6 h exposure to high light (900 µE m^−2^ s^−1^); ** 6 h exposure to UV-B (6.8 W/m^−2^); Canth: canthaxanthin; Zeax: zeaxanthin, *** Sandmann unpublished.
